# A Personalized Signature and Chronotherapy-Based Platform for Improving the Efficacy of Sepsis Treatment

**DOI:** 10.3389/fphys.2019.01542

**Published:** 2019-12-19

**Authors:** Ariel Kenig, Yaron Ilan

**Affiliations:** Department of Medicine, Hadassah-Hebrew University Medical Center, Jerusalem, Israel

**Keywords:** chronobiology, chronotherapy, drug resistance, compensatory mechanisms, sepsis

## Abstract

Sepsis remains a major therapeutic challenge and is associated with a high rate of morbidity and mortality. It is a dynamic condition in which multiple parameters change over time, rendering it difficult to overcome the various injurious responses, which worsen the prognosis in these patients. The prognosis of sepsis is associated with a disbalance of compensatory responses to infectious triggers, part of which can be deleterious. Marked inter- and intra-patient variability characterizes the mechanisms that underlie sepsis progression and determine the response to therapy. In this paper, we review some of the data on the use of chronopharmacological approaches for the treatment of patients with sepsis and discuss the role of the autonomic nervous system in the mechanisms associated with immune response and chronotherapy in these patients. We describe the implementation of an individualized platform that is based on the personalized autonomic nervous system, immune, and chronobiology-derived parameters for generating a patient-tailored therapeutic regimen. The notion of overcoming the deleterious compensatory response in a highly dynamic system in sepsis is presented to ensure an improved response to current therapies.

## Introduction

Sepsis is a life-threatening condition caused by a dysregulated host response to infections and is a leading cause of mortality worldwide (Vincent et al., 2014; Fleischmann et al., 2016; Singer et al., 2016). It is also associated with high morbidity among the survivors, with almost 60% of them having at least one episode of re-hospitalization in the first year (Shankar-Hari and Rubenfeld, 2016). Sepsis accounted for $23.7 billion (6.2%) of the United States National Inpatient Hospital cost in 2013, making it the most expensive medical condition to treat (Torio and Moore, 2006). In 2016, the definitions and criteria of sepsis have been revised placing the inflammatory host response as the pivotal cause of multi-organ failure (MOF) (Singer et al., 2016). This process contributes to the absence of response toward the current therapies leading to a high rate of morbidity and mortality (Iskander et al., 2013; Gotts and Matthay, 2016). Merely a few interventions in the management of sepsis have strong supporting data (Rhodes et al., 2017). Deleterious prognosis in these patients might be partly related to the lack of methods for personalizing the therapies in a highly dynamic system (Pinheiro da Silva and Cesar machado, 2015).

In this article, we review some data on the use of chronopharmacological approaches for the treatment of patients with sepsis and describe the establishment of a personalized quantified signature and chronobiology-based platform as a mean of improving the response to therapies in patients with sepsis.

### Uncontrolled Compensatory Response to Sepsis Contributes to the Deterioration of the Disease

The prognosis of sepsis is determined by a balance between the pro- and anti-inflammatory responses. During sepsis, infectious insults lead to the activation of the innate immune system, and alteration of macrophages, lymphocytes, platelets, and complement system function. These are associated with the release of pro-inflammatory mediators, which assist in the elimination of pathogens. Further, induction of tissue regeneration may also mediate tissue damage. Local tissue injury and inflammatory responses are associated with a “spill-over” phenomenon of chemotactic mediators and pro-inflammatory cytokines that recruit monocytes, lymphocytes, and polymorphonuclear leukocytes (PMNs). These cells secrete activating vasoactive mediators, stimulate platelets and the coagulation cascade, and further increase the vascular permeability. This process leads to systemic pro-inflammatory response syndrome (SIRS). In turn, SIRS leads to a vicious cycle wherein an increase in the vascular permeability augments tissue injury (Assinger et al., 2019). However, in response to a stimulus, innate immune cells can either boost the response as a part of the trained immunity or diminish it and induce immune tolerance. SIRS leads to the activation of counter-regulatory homeostatic mechanisms or compensatory anti-inflammatory syndrome (CARS) (Osuchowski et al., 2006; Okazaki and Matsukawa, 2009).

The clinical elements suggestive of CARS include cutaneous anergy, leukopenia, and susceptibility to infections (Ward et al., 2008). CARS is characterized by impaired cellular immunity, lymphocyte dysfunction, monocyte deactivation, defective antigen presentation, and reduced proliferative and pro-inflammatory cytokine production accompanied by lymphocyte apoptosis, downregulation of monocyte HLA-DR expression, and monocyte deactivation. Macrophage dysfunction in CARS is associated with attenuated pro-inflammatory and enhanced anti-inflammatory cytokine profiles, including interleukin-4 (IL-4), IL-10, tumor growth factor beta (TGFb), and prostaglandin E2. Anti-inflammatory mediators inhibit IKK-b- and NF-kB-dependent pro-inflammatory gene transcription and promote apoptosis of activated macrophages. These factors contribute to systemic immune suppression and susceptibility to infection.

The balance between the opposing systemic pro- and anti-inflammatory responses (SIRS and CARS) is vital for determining the prognosis in sepsis. The two opposing processes occur simultaneously (Neunaber et al., 2011; Novotny et al., 2012; Magrone and Jirillo, 2019). A predominance of the pro-inflammatory response is considered to be associated with early mortality (Hotchkiss et al., 2013). SIRS is associated with a worse prognosis, independent of infection. Over time, the balance tilts toward the anti-inflammatory response which is associated with immune suppression, recurrent infections, sepsis, and MOF (Novotny et al., 2012; Parlato and Cavaillon, 2015). Taken together, these data suggest that the uncontrolled compensatory response to sepsis may contribute to the clinical deterioration and worsening of the prognosis.

### The Circadian Rhythm Plays a Role in the Pathogenesis of Sepsis

Humans have developed an endogenous timing system that optimally synchronizes the physiological processes and behavior with the environmental stimulus (Morris et al., 2012). The central pacemaker functions at the suprachiasmatic nucleus (SCN) and is occasionally synchronized with clock genes in the peripheral tissues including immune cells. The hierarchical control from the central to the peripheral clocks involves endocrine, metabolic, immune, and mitochondrial responses (Acuna-Castroviejo et al., 2017). Several clock genes control the periodicity of circadian rhythms including brain and muscle ARNT-like 1 (BMAL1)/circadian locomotor output cycle protein kaput (CLOCK), Period 1 (Per1), Period 2 (Per2), and Cryptochrome (CRY) (Truong et al., 2016).

Chronobiology or the regulation of the biological systems by the circadian rhythm is required for the proper functioning of cells and tissues (Mandal et al., 2010; Truong et al., 2016). Chronobiology is also used as a tool for better-timed drug delivery, termed chronotherapy (Mandal et al., 2010). Chronotherapy is based on linking the absorption, metabolism, and elimination of drugs to the circadian patterns (Dallmann et al., 2014). It may improve the efficacy and reduce the toxicity of chronic medications (Ozturk et al., 2017). In addition, the genes for enzymes involved in all stages of liver detoxification are rhythmically expressed (Dallmann et al., 2014).

Altered circadian rhythm or chronodisruption is associated with alterations of the immune response, immunosenescence, impairment of energy metabolism, and reduction of the pineal and extra-pineal melatonin production (Acuna-Castroviejo et al., 2017). Diverse disease states, including myocardial infarction (MI), asthma, and temporal epilepsy are associated with alterations of the circadian rhythms, thereby influencing the severity of symptoms and the risk of mortality (Litinski et al., 2009). The frequency of MI and sudden cardiac death is higher during the morning hours (Muller et al., 1985; Willich, 1990). This corresponds to an increased platelet aggregability in response to adenosine diphosphate and epinephrine in a similar time frame (Tofler et al., 1987). Circadian rhythm disruption increases the risk of cancer. A study among 78,586 women found an increased risk of colon cancer among night shift workers (Schernhammer et al., 2003). Similar results were described in patients with prostate and breast cancers (Krstev et al., 1998; Hansen, 2001).

The inflammatory response to various triggers also follows a circadian pattern. Cytokines and hormones secretion manifest significant diurnal variations in the plasma concentration (Scheff et al., 2010). A day-night difference in the acute phase response to endotoxemia was shown in healthy human volunteers with a more pronounced inflammatory response during the night (Alamili et al., 2014b). The levels of malondialdehyde and IL-10 were higher during the day, whereas the levels of tumor necrosis factor-alpha (TNFα), IL-6, sTNF-RI, sTNF-RII, and IL-1Ra were higher during the night (Alamili et al., 2014b). Being a dynamic system, both peripheral and central body clocks are entertained by new challenges such as during infections.

Disrupted circadian rhythm has been described in sepsis affecting the response to triggers including infections (Coiffard et al., 2019). Circadian misalignment impacts disease severity, treatment response, and prognosis. Serum levels of IL-6, TNFα, high mobility group box 1, IL-1α, IL-9, eotaxin, and granulocyte colony-stimulating factor were increased in daytime-fed mice with sepsis (Oyama et al., 2014). Murine macrophages subjected to serum shock to synchronize circadian rhythms and exposed to bacteria from septic mice demonstrated fluctuations in IL-6 production. This fluctuation was mediated by a toll like receptor 2 (TLR2)-dependent mechanism (Heipertz et al., 2018). Sepsis patients manifested impaired circadian rhythms of clock genes, cortisol, and cytokines, further comprising the normal oscillatory function. This led to the further deterioration of the disease, increasing the severity of inflammation, and worsening the prognosis of sepsis (Billings and Watson, 2015; Truong et al., 2016).

Reciprocal relationships between the circadian genes and the immune system have been shown. Disruption of the circadian clock genes induces pro-inflammatory mediators, which further alters the clock. Lipopolysaccharide (LPS) increases serum TNFα levels and reduces Per1 and Per2 gene expression (Cermakian et al., 2014). The core clock proteins BMAL1, CLOCK, and reverse-erythroblastosis virus α (REV-ERBα) control immune response (Curtis et al., 2014). BMAL1:CLOCK heterodimer regulates TLR9 expression and reduces the expression of the inflammatory monocyte chemokine ligand CCL2 (Curtis et al., 2014). Circadian expression of the CLOCK gene product Per2 is altered in the SCN of post-septic mice (O’Callaghan et al., 2012). Per2(m/m) mice have shown a down-regulated circadian immune response to LPS. A chronopharmacological lethal effect of LPS was associated with a time-dependent increase in serum TNFα along with increased Per2 gene expression in ZT12-18. Increased apoptosis reflected by a higher Bax mRNA expression level was noted at 8 and 26 h after LPS injection (Yamamura et al., 2010). The serum levels of corticosterone (CORT), which plays a role in immune suppression, are increased in Per2(m/m) mice following LPS administration, and it correlated with longer survival (Wang et al., 2015). Uncoupling of peripheral and master clock gene rhythms by reversed feeding exacerbates inflammatory responses. Daytime feeding was found to induce clock gene uncoupling augmenting inflammatory cytokines leading to a high mortality rate. In a model of daytime-fed mice with sepsis, phase inversion of the clock gene expression was noted in the liver, with a lower survival rate than that in the nighttime-fed mice (Oyama et al., 2014).

The risk of sepsis was suggested to follow a diurnal variation (Kizaki et al., 2015). Mice develop sepsis more rapidly when the disease is induced in the nighttime than the daytime. Mice with a mutated Per2 gene had a similar outcome when sepsis was induced at both times (Heipertz et al., 2018). Studies have shown that light affects immunity, and various neurophysiologic pathways are maximally entrained by the blue spectrum (Lewis et al., 2018). In an animal model of sepsis, the exposure to bright blue light has enhanced the bacterial clearance from the peritoneum and reduced bacteremia and systemic inflammation. The effect was associated with an increased cholinergic tone which augmented tissue expression of the nuclear orphan receptor REV-ERBα (Lewis et al., 2018).

The confinement of sepsis patients in intensive care units (ICU) imposes environmental constancy throughout the day and night leading to further chronodisruption. It is associated with sleep impairment with a pro-inflammatory trajectory (Madrid-Navarro et al., 2015). The daily light/dark (LD) cycle impacts the recovery from sepsis. Circadian cues provided by the LD cycle has improved survival in an animal model of sepsis. Removal of these cues by constant dark increases the early appearance and incidence of a hormonal response pattern and is associated with a lethal outcome (Carlson and Chiu, 2008).

Immune tolerance or “sepsis-induced immunosuppression” is typical for sepsis survivors and is characterized by a hypo-responsiveness of the immune system. This condition renders the host vulnerable to a persisting infection or the occurrence of secondary opportunistic infections leading to an increased mortality rate (Bomans et al., 2018). Prior sepsis alters the responsivity of the circadian system to subsequent immune challenges. LPS-induced sepsis shapes the response to subsequent administration of lower dosages of LPS in mice (Meneses et al., 2018). Testing the responsiveness of the circadian system of mice to LPS showed that while in control animals LPS induced a significant phase delay of the behavioral rhythm, it did not occur in post-septic animals (Castanon-Cervantes et al., 2010). Post-septic animals showed elevated expression of immediate early genes c-Fos and early growth response protein 1 in the hippocampus but not in the SCN supporting the notion that sepsis affects the molecular responses to subsequent immune challenges (Anderson et al., 2015).

Melatonin follows a stable circadian rhythm and regulates the sleep-wake cycle by interacting with the neuroendocrine and immune systems. Sepsis and drugs disrupt the circadian secretion of melatonin compromising the immune response. Impaired circadian melatonin secretion in sepsis patients led to abnormalities in the urinary level of 6-sulfatoxymelatonin (6-SMT), the major melatonin metabolite (Mundigler et al., 2002; Verceles et al., 2012). The excretion of 6-SMT increased and it correlated with the degree of the immune response, oxidative status, sequential organ failure assessment (SOFA) score, and procalcitonin level in patients with sepsis. This was associated with a blunted expression of the clock genes bmal1, per2, clock, and cry1. The innate immune cytokines IL-1β, IL-6, IL-8, TNFα, and IL-10 and the oxidative stress responses were increased in these patients (Acuna-Fernandez et al., 2019). Administration of melatonin normalized the clock and the innate response, thereby reducing the pro-inflammatory cytokine levels on daytime endotoxemia as compared to placebo, whereas, it did not affect the nighttime cytokine levels (Alamili et al., 2014a). Melatonin administration increased the survival time in the murine sepsis model, an effect that was blunted by the double knockout of melatonin receptors MT1/MT2 (Fink et al., 2014). Overall the data support an association between the disruption of the circadian rhythm and the pathogenesis of sepsis and the response to therapy.

### Variability Characterizes the Biological Systems, and Heart Rate Variability Reflects the Function of the Autonomic Nervous System in Health and Diseases

Variability is inherent to all biological systems from the cellular to the whole organ levels and is a part of the function of the normal tissues (Ilan, 2019a,b,c,d,e). Loss of variability implies loss of regulation and is associated with disease states and poor prognosis (Costa et al., 2014; Singh et al., 2018). The ANS affects chronobiology in various biological systems (Thosar et al., 2018; Baschieri and Cortelli, 2019; Becker et al., 2019). The ANS, through shifting the balance between its sympathetic and parasympathetic branches, modulates the length of the heart cycle. Heart rate variability (HRV) signifies the irregularities of the intervals between adjacent heartbeats and is a quantitative marker of the autonomic nervous system (ANS) balance (Kleiger et al., 1987; Vanderlei et al., 2009; Shaffer and Ginsberg, 2017). HRV reflects the function of the pacemaker cells clocks in the SAN (Yaniv et al., 2015). HRV exhibits diurnal variation, with the parasympathetic parameters peaking at nighttime and plateauing at daytime (Bonnemeier et al., 2003). The parasympathetic activity dominates at rest and exerts its effect more rapidly than the sympathetic nerves (Shaffer et al., 2014). HRV measures are sleep-state dependent, implying that the circadian and stage-specific processes influence the ANS modulation of HR (Boudreau et al., 2013).

Heart rate variability indices are divided generally to time domain, frequency domain, and non-linear measurements (Shaffer and Ginsberg, 2017). Time-domain measures describe the variability of RR interval length. The simplest time domain index is the standard deviation (SD) of normal RR intervals (SDNN), which due to the negligible fluctuation in the atrioventricular conduction time, is regarded as a measure of the sinoatrial node (SAN) cycle length fluctuation (Zaza and Lombardi, 2001). This fluctuation reflects the systemic autonomic tone. Frequency domain measures reflect how power distributes as a function of the frequency, it includes the ultra-low frequency, very low frequency (VLF), low frequency (LF), and high frequency (HF) (Malliani et al., 1991; Shaffer and Ginsberg, 2017). While broad evidence supports the vagal origin of the HF component, the link of the LF component to sympathetic activity is controversial (Goldstein et al., 2011; Reyes del Paso et al., 2013). The LF band is controlled by the vagus nerve providing information on blood pressure control mechanisms, such as the baroreflex (Reyes del Paso et al., 2013). The VLF is controlled by multiple factors including the parasympathetic tone, the renin-angiotensin-aldosterone system, and thermoregulation (Berntson et al., 1997; Taylor et al., 1998).

Heart rate variability measures, mean RR, SDNN, HF, LF, and LF/HF are reduced among night shift workers (Lee et al., 2015). Healthy subjects experience a decline in all the HRV measures with age (Umetani et al., 1998). Lower HRV is associated with higher all-cause mortality in middle-aged and elderly men. A single SD decrement in the LF power is associated with all-cause mortality (Tsuji et al., 1994). HRV measures are linked to cardiovascular risk factors (Felber Dietrich et al., 2006; Thayer et al., 2010). Hypertensive patients have lower LF than normotensive patients, and non-HDL cholesterol levels are associated with lower total power, HF, and LF (Felber Dietrich et al., 2006). In a study on 11,654 patients, a lower HRV was associated with the development of coronary heart disease in diabetes patients (Liao et al., 2002). A doubled risk of mortality was reported in patients with diabetes, hypertension, or cardiovascular diseases with reduced HRV (Gerritsen et al., 2001). Lower SDNN values carry an increased risk of mortality post-MI (La Rovere et al., 1998), and low HRV has a prognostic value in patients with congestive heart failure and non-ischemic dilated cardiomyopathy (Nolan et al., 1998; Rashba et al., 2006; Thayer et al., 2010).

The ANS may also be associated with the regulation of the immune system, and HRV has been shown as a marker of inflammation (Habek, 2019; Koopman et al., 2017). The ANS and immune system have a reciprocal influence on each other during inflammation. Reduced HRV is independently related to C-reactive protein and white blood count levels in healthy individuals (Sajadieh et al., 2004). Asthma severity correlated with the LF band, HF band, and HRV SD (mean SD for all RR intervals) (Kazuma et al., 1997). Controlled asthma is associated with enhanced parasympathetic modulation and higher HRV compared to uncontrolled asthma (Lutfi, 2015). Studies on systemic lupus erythematosus (SLE) patients reported a correlation of HRV with the cytokine levels and the clinical status (Thanou et al., 2016; Matusik et al., 2018). Similarly, HRV has been found to predict response to therapy in rheumatoid arthritis (RA) (Holman and Ng, 2008). It was suggested that combining HRV in the therapeutic regimens of RA could save $8 billion and over 100,000 quality-adjusted life years over a 10-year period (Zimmermann et al., 2018).

The utility of HRV has been also proposed in malignant diseases. A systemic meta-analysis concluded that there is a role of HRV in predicting the survival of patients with cancer (Zhou et al., 2016). HRV correlates with tumor size, infiltration, progression, and metastasis formation in patients with gastric cancer (Hu et al., 2018). LF/HF ratio is a surrogate marker for pain management in cancer patients (Masel et al., 2016). These represent the dysregulation of the parasympathetic system presenting as a reduced HF power in patients with chronic pain (Tracy et al., 2016).

### ANS and HRV-Related Parameters Are Altered in Sepsis: A Role of Chronobiology in the Pathogenesis of the Disease and Response to Therapy

Systemic inflammation induces alterations in the sympathetic-vagal balance in the ANS. The rhythmic recruitment of leukocytes in the tissues is regulated by signals received from the sympathetic nervous system delivered by adrenergic nerves (Scheiermann et al., 2012). Leukocyte adhesion and migration in the bone marrow and the skeletal-muscle microvasculature peak at night (Scheiermann et al., 2012). Migratory oscillations, which are altered by jet lag, are implemented by perivascular sympathetic nervous system fibers, which activate beta-adrenoreceptors on non-hematopoietic cells. These signals lead to circadian fluctuations in the expression of endothelial cell adhesion molecules and chemokines, which contribute to the development of sepsis (Scheiermann et al., 2012). Vagal signaling through alpha7 nicotinic acetylcholine receptor inhibits the production of pro-inflammatory cytokines (Wang et al., 2016). There is however, an uncertainty regarding the vagal mediated anti-inflammatory reflex as a response automatically triggered by inflammation. An electrical connection from the vagus to the spleen, which mediates a major proportion of the systemic inflammatory response to endotoxemia, has not been found (Martelli et al., 2014).

The dysfunction of the ANS has been described in sepsis (Badke et al., 2018). Both alterations in respiratory rate, systolic blood pressure, and temperature, along with HRV, can serve as parameters to be followed. Increased HR was noted in septic mice in a time-dependent manner, and was noted mainly at ZT6, 12, and 18. The HR alterations correlated with an increased TNFα mRNA expression by LPS in the heart. Further, HF power rhythm coincided with the levels of urinary 6-SMT, the melatonin metabolite (Boudreau et al., 2011). In sepsis patients, alpha7 mRNA levels correlated directly with vagal HRV patterns and inversely with inflammation level and outcome (Cedillo et al., 2015). In a randomized study in healthy men, LPS was administered in a day or night visit. Endotoxemia during both night and day resulted in a depression of HF, LF, and SDNN intervals along with an increased ratio of LF/HF and mean HR. However, nighttime endotoxemia was associated with a more pronounced depression of LF, HF, and SDNN, and a more pronounced increase in the ratio of LF/HF and mean HR, suggesting that changes in HRV mediated by endotoxemia show diurnal variation (Alamili et al., 2015).

Changes in HRV maybe associated with impending septic shock and increased mortality in sepsis patients (Chen and Kuo, 2007; de Castilho et al., 2018). A systemic review of clinical studies suggested a reduction in HRV parameters in non-surviving sepsis patients (de Castilho et al., 2018). In one study, after adjusting for SOFA and acute physiology and chronic health evaluation II (APACHE II) scores, an SDNN of ≤17 ms was associated with a hazard ratio of 6.3 for increased mortality (de Castilho et al., 2017). Measurement of mean RR interval and detrended fluctuation analysis α2 (DFA-α2) alongside age, respiratory rate, and systolic blood pressure created a predictive model for severe sepsis (Samsudin et al., 2018). Combining HRV with other laboratory tests generated a predictive model that could improve the current scores (Barnaby et al., 2019). Adding DFA-α2 to qSOFA was suggested as a means for improving the accuracy for predicting mortality (Prabhakar et al., 2019). In a recent study, machine learning models were applied to HRV measurements, resulting in improved 30-day mortality prediction (Chiew et al., 2019). While most studies on the prognostic yield of HRV were not based on the Sepsis-3 criteria, they demonstrate the potential of HRV-based prognostic decision-making regarding treatment escalation and the need for ICU.

However, reduced HRV is associated with end-organ responsiveness of cardiac pacemaker cells or with their functional dynamics, rather than altered activity of the ANS. The reduction in HRV sepsis might reflect modulation of inflammatory mediators on cardiac pacemaker cells, rather than alteration of the ANS signaling itself (Gholami et al., 2012; Mazloom et al., 2014). Endotoxin challenge was described to induce hyporesponsiveness of spontaneously beating atria to cholinergic stimulation but not in chronotropic response to chronotropic adrenergic stimulation (Gholami et al., 2012). Furthermore, previous studies showed alteration in HR dynamics in denervated isolated hearts, suggesting a reduced controllability of cardiac rhythm following endotoxemia (Mazloom et al., 2014). The intrinsic response of pacemaker cells to the inflammatory process might be a player in the mechanism of HRV blunted in sepsis. Adding respiratory rate, and temperature may assist in overcoming the difficulty of using HRV as a sole measure for the ANS in septic patients.

### Establishing Personalized Signatures and Chronotherapy-Based Platform for Improving the Efficacy of Treatment in Sepsis

Sepsis is a highly dynamic system in which multiple factors determining the course of the disease and response to therapy are involved. Marked inter- and intra-patient changes occur in sepsis, as well as in the compensatory response to the infectious and other triggers in patients. These make it difficult to design a “one bullet for all” therapeutic regimen.

The association of sepsis prognosis with immune response, chronobiology, ANS, and HRV, respiratory rate, systolic blood pressure, and temperature, indicates that these parameters should be considered in the individualization of sepsis therapy. This association underlies the platform for improving the response to therapy in sepsis in a personalized manner. Dysregulation of the inflammatory response contributes to the mechanism of sepsis. Inflammation may lead to recovery and homeostasis. However, in cases where the compensatory response is inadequate, a persistent inflammatory state remains and is associated with a bad prognosis (Scheff et al., 2013). Computational systems biology is used for the development of methods for dealing with these complex networks of interacting pathways. Specifically, attempts were made to include biological rhythms and adjustments of these rhythms along with the physiologic variability into therapeutic regimens. While somewhat difficult to follow, scales based on oscillations in the autonomic activity developed from short-term variability in circadian rhythms in immunomodulatory hormones were suggested as parameters for these algorithms (Scheff et al., 2013).

The establishment of a new platform for improving the prognosis of sepsis patients is based on comprising the following three steps into a distinct dosing regimen. In the first step, chronotherapy-based regimens are implemented into the treatment regimen. HRV, which reflects the ANS tone, can serve as a chronobiological marker for directing therapy during sepsis. These are performed based on regular therapeutic regimens that are associated with chronobiology in a non-personalized way. The use of alternate dosing regimens was suggested to improve the therapeutic effects of chronic medications overcoming the loss of response, which is common in patients with chronic diseases (Strik et al., 2019).

In the second step, signatures of variability, such as HRV and immune-derived signatures which are linked to the pathogenesis of the disease and response to therapy are implemented in the treatment algorithm. These are suggested to overcome the adaptation of the body system, manifested by a deleterious compensatory response and loss of the effects of drugs. Implementing randomness in the dosing and timing of therapeutic maneuvers including mechanical ventilation parameters and medications are introduced in a non-individualized way. Preliminary attempts in this direction can be seen, for example, in the application of HRV for prediction of mechanical ventilation weaning results (Huang et al., 2014) and real-time monitoring tool for nutritional manipulation in sepsis (Papaioannou and Pnevmatikos, 2019).

In the final step, individualization of chronotherapy and variability measures are being implemented in the treatment algorithm in a continuous dynamic way, which works in a closed loop and responds to individual alterations recorded from each patient. Personalized chronotherapy and variability-based signatures can be quantified and implemented into the treatment algorithm. A multi-dimensional sepsis management personalized model incorporates HRV alongside immune signatures, laboratory parameters, imaging findings, and clinical parameters.

**FIGURE 1:**
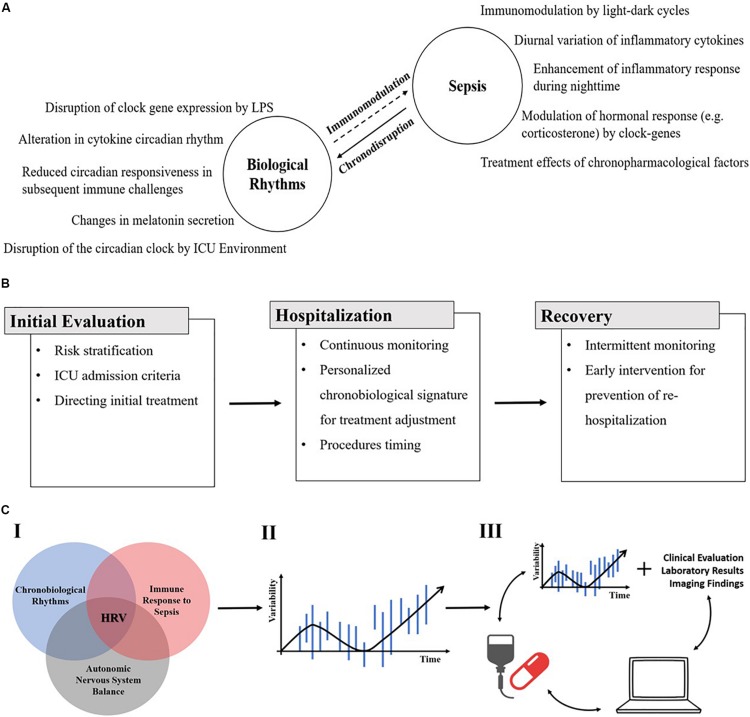
**(A)** A schematic presentation of the bidirectional effect of sepsis and chronobiology. Examples of biological rhythms influence on the inflammatory response during sepsis (right side) and of the alteration of those rhythms (chronodisruption) by the inflammatory process itself (left side) (ICU, intensive care unit). **(B)** A schematic representation of the potential use of HRV from the diagnosis and risk stratification through personalizing the therapy to match relevant chronobiological patterns, and finally in the post-sepsis observation period (HRV, heart rate variability). **(C)** A schematic representation of establishing a platform that implements different signatures that are relevant to sepsis, including circadian rhythm, immune-, and ANS-based parameters (I). These are implemented into a variability-based therapeutic regimen (II). Finally, the generation of a patient-based closed loop, which responds to the continuous changes is implemented (III) (HRV, heart rate variability).

Figure 1 shows a schematic representation of the potential implementation of the new platform for the improvement of the response to therapy in patients with sepsis. Panel A shows the bidirectional effects of sepsis and chronobiology. Biological rhythms impact the inflammatory response during sepsis and alterations of these rhythms (chronodisruption) by the inflammatory process both contribute to the pathogenesis of the disease and the response to therapy. Panel B shows the potential use of HRV from the diagnosis and risk stratification through personalizing the therapy to match relevant chronobiological patterns, and finally in the post-sepsis observation period. Panel C shows a schematic representation of establishing a platform that implements different signatures that are relevant to sepsis, including circadian rhythm, immune, and ANS-based parameters. These are implemented into a variability-based therapeutic regimen based on which the generation of a patient-based closed loop, which responds to the continuous changes, is implemented.

The implementation of the proposed platform faces some challenges. First, additional researches on the mechanisms of ANS role in sepsis and on HRV alteration during sepsis are needed to firmly establish the theoretical basis of the proposed platform. Second, on the practical level, continuous HRV measurement in hospital setting might be challenging and requires interfaces with current hospital monitoring systems. However, novel wearable monitoring devices being developed provide accessible monitoring of HRV among other measures. This can enable the integration of other continuous measures effected by the ANS, such as respiratory rate and systolic blood pressure in the suggested personalized approach.

## Conclusion

In summary, improving the therapy of patients with sepsis is a major unmet need. Being a highly dynamic condition in which multiple parameters continuously change over time, it is difficult to overcome the compensatory responses that are sometimes deleterious and contribute to the worsening of the prognosis. Moreover, the marked inter- and intra-patient variability, which characterizes both the pathogenic mechanisms and the response to therapy, necessitates the use of a model that could individualize the therapeutic regimens. Ongoing studies will determine the ability to implement individualized platforms based on biological variability parameters, ANS-based sensors, immunity, and chronobiology-derived signatures for generating a patient-tailored therapeutic regimen for improving the response to current drugs and therapeutic maneuvers.

## Author Contributions

Both authors wrote the manuscript.

## Conflict of Interest

YI is the founder of Oberon Sciences and is a consultant of Teva, ENZO, Protalix, Betalin Therapeutics, Immuron, SciM, Natural Shield, Oberon Sciences, Tiziana Pharma, Plantylight, and Exalenz Bioscience. The remaining author declares that the research was conducted in the absence of any commercial or financial relationships that could be construed as a potential conflict of interest.

## Abbreviations

%RR50: proportion of cycles with RR difference > 50 ms
6-SMT: 6-sulfatoxymelatonin
ANS: autonomic nervous system
APACHE II: acute physiology and chronic health evaluation II
BMAL1: brain and muscle ARNT-like 1
CARS: compensatory anti-inflammatory response syndrome
CLOCK: circadian locomotor output cycle protein kaput
CORT: corticosterone
CRY1: cryptochrome1
DFA-2 α: detrended fluctuation analysis α 2.
HF: high frequency
HRV: heart rate variability
ICU: intensive care unit
IL: interleukin
LD: light/dark
LF: low frequency
LPS: lipopolysaccharide
MI: Myocardial Infraction
MOF: multi-organ failure
Per1: period 1
Per2: Period 2
PMNs: polymorphonuclears
RA: rheumatoid arthritis
REV-ERB α: reverse-erythroblastosis virus α
SAN: sinoatrial node
SCN: suprachiasmatic nucleus
SD: standard deviation
SDNN: standard deviation of normal RR intervals
SIRS: systemic inflammatory reaction syndrome
SLE: systemic lupus erythematosus
SOFA: sequential organ failure assessment
TGF β: tumor growth factor beta
TLR: toll like receptor
TNF α: tumor necrosis factor α
VLF: very low frequency
